# A novel tracer for in vivo optical imaging of fatty acid metabolism in the heart and brown adipose tissue

**DOI:** 10.1038/s41598-020-68065-4

**Published:** 2020-07-08

**Authors:** Marcello Panagia, Jing Yang, Eric Gale, Huan Wang, Ivan Luptak, Howard H. Chen, Dakshesh Patel, Dominique Croteau, David Richard Pimentel, Markus Michael Bachschmid, Wilson S. Colucci, Chongzhao Ran, David E. Sosnovik

**Affiliations:** 1 Cardiovascular Medicine Section, Department of Medicine, Boston University Medical Center, Boston, MA USA; 2 Cardiovascular Research Center, Massachusetts General Hospital, Boston, MA USA; 3 Martinos Center for Biomedical Imaging, Department of Radiology, Massachusetts General Hospital, Boston, MA USA; 4 Vascular Biology Section, Department of Medicine, Boston University School of Medicine, Boston, MA USA; 5 Boston University School of Medicine, Whitaker Cardiovascular Institute, 650 Albany St, Boston, MA 02118 USA

**Keywords:** Optical imaging, Energy metabolism

## Abstract

Multiplexed imaging is essential for the evaluation of substrate utilization in metabolically active organs, such as the heart and brown adipose tissue (BAT), where substrate preference changes in pathophysiologic states. Optical imaging provides a useful platform because of its low cost, high throughput and intrinsic ability to perform composite readouts. However, the paucity of probes available for in vivo use has limited optical methods to image substrate metabolism. Here, we present a novel near-infrared (NIR) free fatty acid (FFA) tracer suitable for in vivo imaging of deep tissues such as the heart. Using click chemistry, Alexa Fluor 647 DIBO Alkyne was conjugated to palmitic acid. Mice injected with 0.05 nmol/g bodyweight of the conjugate (AlexaFFA) were subjected to conditions known to increase FFA uptake in the heart (fasting) and BAT [cold exposure and injection with the β_3_ adrenergic agonist CL 316, 243(CL)]. Organs were subsequently imaged both ex vivo and in vivo to quantify AlexaFFA uptake. The blood kinetics of AlexaFFA followed a two-compartment model with an initial fast compartment half-life of 0.14 h and a subsequent slow compartment half-life of 5.2 h, consistent with reversible protein binding. Ex vivo fluorescence imaging after overnight cold exposure and fasting produced a significant increase in AlexaFFA uptake in the heart (58 ± 12%) and BAT (278 ± 19%) compared to warm/fed animals. In vivo imaging of the heart and BAT after exposure to CL and fasting showed a significant increase in AlexaFFA uptake in the heart (48 ± 20%) and BAT (40 ± 10%) compared to saline-injected/fed mice. We present a novel near-infrared FFA tracer, AlexaFFA, that is suitable for in vivo quantification of FFA metabolism and can be applied in the context of a low cost, high throughput, and multiplexed optical imaging platform.

## Introduction

In organs with high metabolic rates, such as the heart and brown adipose tissue (BAT), the ability to quantify substrate utilization is crucial for understanding dynamic pathologic states^1–3^. Moreover, simultaneous, multiplexed measurements are essential in tissues that can quickly alter substrate utilization depending on the pathophysiological context and hormonal milieu^2,4–7^. However, molecular imaging of energy substrate utilization has been challenging. The ideal platform to evaluate substrate utilization, particularly in pre-clinical models, should allow for multiple substrates and metabolic pathways to be concurrently imaged in a high throughput and low-cost fashion with adequate sensitivity to discriminate changes in physiology. Positron emission tomography (PET) can provide high sensitivity and good throughput, but it is limited by cost (both equipment and radiotracer synthesis costs) and by its limited ability to multiplex readouts^7,8^. Although significant research interest has been devoted to developing multi-isotope PET imaging, currently, in both clinical and pre-clinical use, a PET approach usually allows for only one tracer to be imaged at one time^9,10^. Similarly, hyperpolarized 13C MR spectroscopy, using substrates such as 13C labeled pyruvate and 13C butyrate, provides the ability to trace downstream metabolic products and assess metabolic flux, but it also suffers from low throughput, inability to multiplex, high cost and will require further advances before becoming a routine research tool^11,12^. Therefore, the ability to simultaneously interrogate multiple energy substrates in vivo poses a continued challenge.

An optical approach for imaging energy metabolism, although challenging, fulfills many of the criteria for a robust pre-clinical platform. Our group has previously shown that it is possible to quantify fatty acid uptake and glucose uptake in multiple tissues, simultaneously, using a commercially available boron-dipyrromethene (Bodipy) conjugated to palmitate with the concomitant injection of 2-deoxy-2-[18F]fluoroglucose (^18^FDG)^13^. In this initial study, ex vivo fluorescence reflectance imaging was used to assess fatty acid uptake and Cerenkov luminescence was used to measure glucose uptake simultaneously. Although the emission spectrum of the Bodipy conjugate necessitated ex vivo imaging for deeper tissues, Cerenkov luminescence of ^18^FDG uptake in deep structures, such as the heart, was possible. Moreover, recent studies have shown that Cerenkov luminescence imaging is comparable to 3-D PET for quantification^14^.

Other methods to assess lipid metabolism with optically active probes have either been spectrally limited and mostly confined to in vitro applications^15–17^ or have required more complicated, genetically engineered, mouse models and thus have not been widely adopted^18^. Therefore, in vivo optical imaging of free fatty acid uptake continues to have practical deficiencies and an inexpensive approach that can be applied to any preclinical rodent strain without genetic modification is still lacking. Here, we describe a straightforward approach using click chemistry to covalently couple a physiologically relevant free fatty acid to a fluorochrome (Alexa Fluor™ 647) that emits light in the near-infrared range (NIR). The conjugate (AlexaFFA) was then tested in BAT and cardiac tissue under a variety of physiologic conditions allowing for both ex vivo and in vivo quantification.

## Materials and methods

### Chemical synthesis and verification

#### General

All chemicals and solvents were purchased commercially and used without further purification.

#### Synthesis

The copper-free click reaction between 0.50 mg Click-IT Alexa Fluor 647 DIBO Alkyne (0.33 μmol, based on the molecular weight estimate provided by the vendor; Thermo Fisher Scientific C10408) and 10 molar equivalent of 15-azidopentadecanoic acid (0.94 mg, 3.3 μmol; Thermo Fisher Scientific C10265) was performed in 100 μL of methylsulfonylmethane (dimethyl sulfoxide; DMSO). The reaction was monitored by HPLC to confirm full conversion of the alkyne precursor. The coupling product (AlexaFFA) was used without further purification.

#### HPLC and MS method

Liquid chromatography-mass spectrometry (LC–MS) was performed using an Agilent 1,100 Series apparatus with an LC/MSD trap and Daly conversion dynode detector with UV detection at 254 nm. The method used on these systems is as follows: Luna C18 column (100 × 2 mm 100 Å); eluent A: 10 mM ammonium acetate in water, eluent B: 95% acetonitrile/10% 10 mM ammonium acetate in water; gradient: 5% B for 1 min, 5% B to 95% B over 10 min, 95% B for 1 min, 95% B to 5% B for 1 min, then 5% B for 2 min; flow rate 0.7 mL/min.

### Animal usage and experimental protocols

All procedures in this study were performed in accordance with animal protocols approved by the Institutional Animal Care and Use Committee of Boston University and Massachusetts General Hospital. All experimental protocols were approved by Boston University and Massachusetts General Hospital Research Compliance Comittees.

### In vitro kinetics

H9C2 cells were seeded at a density of 1 × 10^5^ cells per well in a 24-well plate. After 24 h of serum starvation (minimal DMEM without serum), time and concentration dependent uptake of AlexaFFA was estimated using SORP 4 Laser BD Fortessa flow cytometer (BD Biosciences, Franklin Lakes, NJ, USA). For time-course experiments, serum starved cells were exposed to 1 μM AlexaFFA for multiple time-points up to 3 h. Cells were trypsinized, resuspended with PBS and immediately analyzed using flow cytometry to determine the percentage of AlexaFFA positive cells. Similarly, for concentration response experiments, serum starved H9C2 cells were exposed to varying concentrations of AlexaFFA for 2 h, followed by collection of cells and estimation of percent AlexaFFA positive cells as above. Flow cytometry data was analyzed with Flowjo software version 10.

### In vivo blood half-life

Stock 2 mM AlexaFFA in DMSO was mixed with phosphate-buffered saline (PBS) to produce 2 nmol of AlexaFFA in a final volume of 100 μL with a final concentration of DMSO of 4% v/v. The pale blue solution was injected (0.05 nmol/g body weight) via tail vein into 4–6 month old C57BL6J female mice (n = 3). A tail snip was performed and a drop of blood was collected at multiple time points (15 min, 30 min, 1, 6, 24 and 30 h) and added to individual black bottom wells containing 100 μL of PBS. The fluorescence intensity for each time point was quantified using a Tecan Infinite M1000Pro plate reader with an excitation wavelength of 647 nm and an emission wavelength of 668 nm and with an excitation and emission bandwidth of 10 nm.

### Ex vivo optical imaging

To test whether the AlexaFFA tracer responded to the same physiologic stimuli that would result in free fatty acid (FFA) uptake in the heart and brown adipose tissue (BAT), 4–6 month old female C57BL6J mice were injected (0.05 nmol/g body weight) with AlexaFFA by tail vein as described above. The mice were then housed at room temperature with ad libitum access to food and water (n = 7) or housed at 4 °C without access to food but with access ad libitum to water (n = 7). After 30 h, the mice were euthanized, and BAT and heart were harvested. Interscapular BAT was carefully dissected from surrounding white adipose tissue using a dissecting light microscope. Whole hearts were sectioned into short-axis slices (3–4 slices per heart). The tissues were immediately washed in ice-cold PBS and imaged using the IVIS Spectrum fluorescence reflectance imaging system (Perkin Elmer, Boston, MA, USA). The excitation wavelength was 640 nm and the emission wavelength was 680 nm. Other parameters included: binning 4, field of view 13 cm, F-stop 2, and exposure time of 2 s.

Analysis of the optical images was performed using the Living Image v4.5 software package (PerkinElmer, Boston, MA, USA). A region of interest (ROI) was drawn manually around the tissues. Radiant efficiency (radiance/μW/cm^2^) was calculated for AlexaFFA. An equivalently sized ROI was drawn adjacent to the tissue and served as the background signal, which was subtracted from the average radiant efficiency. Data were normalized to tissue weight and the values from simultaneously imaged control animals.

### Microscopy

Freshly isolated heart and BAT samples were embedded in Tissue-Tek optimum cutting compound (OCT) (VWR 25608-930) and slowly frozen using an ethanol and dry ice slurry and stored at − 80 °C. Cryo-sectioning was then performed and 10 μm thin sections were placed on glass slides. Sections were subsequently stained with DAPI (2 μg/mL) (Servicebio Technology LTD, Lot YP190801) and mounted using Hardset Antifade Mounting Medium (Servicebio Technology LTD Lot: 20180422). Fluorescence imaging was then performed with a Panoramic MIDI II (3D HISTECH Ltd). For DAPI an excitation wavelength of 377 ± 50 nm and emission wavelength of 447 ± 60 nm with an exposure time of 3 ms was used. For AlexaFFA, an excitation wavelength of 628 ± 20 nm and emission wavelength of 692 ± 20 nm with an exposure time of 80 ms was used. Images were then analyzed using Image J software (v1.51; National Institutes of Health). Hematoxylin and Eosin (H & E) staining was performed in paraffin-embedded, freshly excised, BAT as previously described^19^.

### In vivo optical imaging

Female nude (COX7 Jackson Labs) mice (6 months old) were injected (0.05 nmol/g body weight) with AlexaFFA by tail vein as described above. The mice were then either injected (1 mg/kg I.V.) with the β3 adrenergic agonist CL 316, 243(CL) and fasted overnight to stimulate FFA uptake in BAT (CL compound) and the heart (fasting) or injected with saline and fed ad libitum. Both groups (n = 6) were housed at room temperature. After 30 h, the mice were anesthetized with isoflurane and in vivo fluorescence reflectance imaging of BAT and the heart was performed using the IVIS Spectrum imaging system (Perkin Elmer). The excitation wavelength was 640 nm and the emission wavelength was 680 nm. Other parameters included: binning 8, field of view 22.4 cm, F-stop 2, and exposure time of 0.5 s. Analyses of optical images were performed as described above for ex vivo optical imaging. Average radiant efficiency was normalized to simultaneously imaged control animals (saline-injected/fed).

### Statistical analysis

Comparison between two groups was performed with either one-way ANOVA or unpaired t test as appropriate. Two-tailed probability values are reported and statistical significance is defined as p < 0.05. Fitting of data was performed using a non-linear regression model with a two-phase decay. Values are reported as mean ± standard deviation (SD). All statistical analyses were performed using Prism v6.0 (Graphpad Software Inc. LaJolla, CA, USA).

## Results

### Chemical synthesis

**Figure 1:**
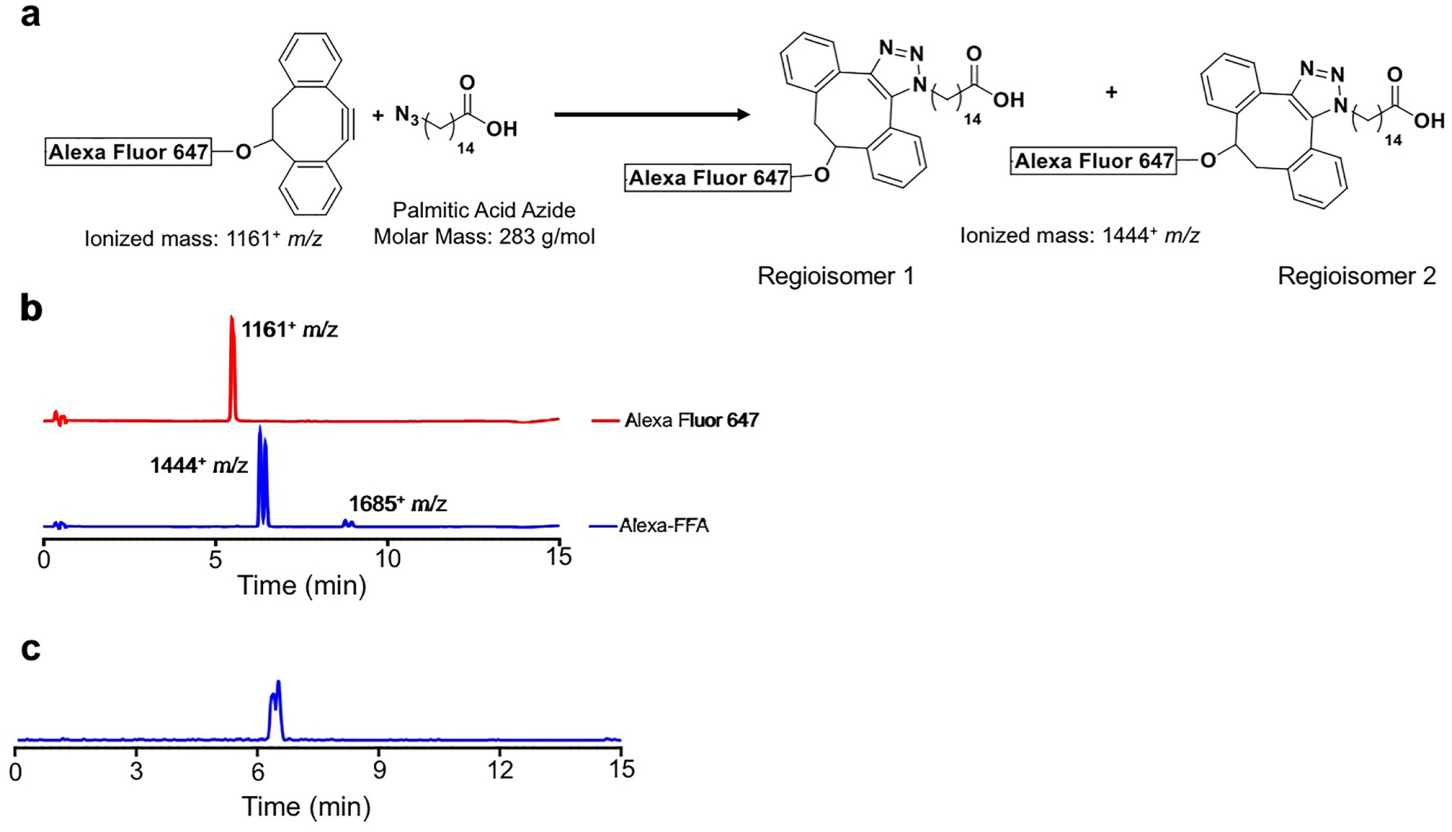
Chemical synthesis and characterization of AlexaFFA. (**a**) Schematic of the coupling reaction between Click-IT™ Alexa Fluor 647 DIBO Alkyne and 15-azidopentadecanoic acid producing two regioisomers. (**b**) LC–MS (UV detection 254 nm) of Alexa Fluor 647 DIBO Alkyne (red line) and the coupling products (blue line) showing a doublet with an ionized mass of 1,444^+^ m/z (regioisomer 1,2) and a by-product with an ionized mass of 1685^+^ m/z. (**c**) Mass spectrometry of the extracted coupling products (m/z = 1,444^+^) showing closely eluting but distinct species.

Figure 1 depicts the reaction scheme between Click-IT Alexa Fluor 647 DIBO Alkyne (shown in generic structure) and 15-azidopentadecanoic acid (palmitic acid azide) producing two coupling products (regioisomer 1 and regioisomer 2). HPLC traces indicated that the ionized mass of Alexa Fluor 647 DIBO Alkyne is 1,161^+^ *m/z* and that of the primary coupling product (94% purity) is 1,444^+^ *m/z* with a minor (by-product) at 1685^+^ *m/z* (6%) (Fig. 1b). Mass spectrometry of the extracted coupling products (*m/z* = 1,444^+^) revealed the two closely eluting species at t_R_ = 6.29 min and 6.45 min which are assigned to the two regioisomeric products of the copper-free click reaction (Fig. 1c).

### In vitro kinetics

**Figure 2:**
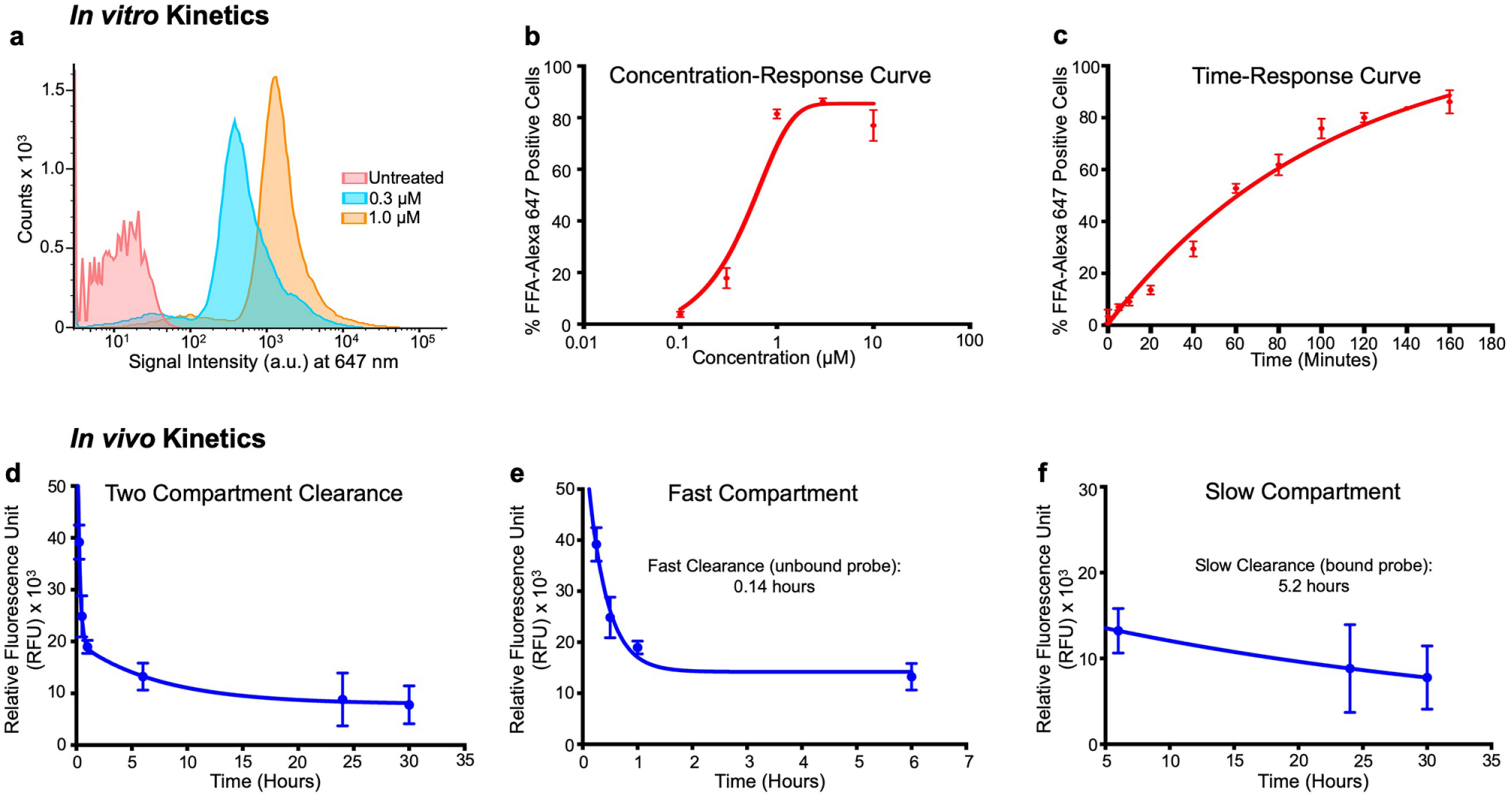
In vitro and blood half-life kinetics of AlexaFFA. Flow cytometry data in H9C2 cells showing (**a**) a representative flow cytometry histogram of concentration-dependent increase in AlexaFFA positive H9C2 cells treated for 2 h which is graphically depicted (**b**) and curve-fitted with one site non-linear fit (r^2^ = 0.96. n = 3). A time dependent uptake of serum-starved cells when exposed to 1 µM AlexaFFA (**c**) and curve fitted with one phase association non-linear fitting (r^2^ = 0.98, n = 3). Blood clearance kinetics (**d**) measured over 30 h after I.V. injection of AlexaFFA showing a two-compartment clearance model. Blood clearance kinetics of AlexaFFA over the first 6 h post-injection (**e**) showing an initial rapid reduction in probe concentration (half-life 0.14 h), likely reflecting the renal elimination of free AlexaFFA. Probe kinetics over the subsequent 24 h (**f**) showing a slower clearance (half-life 5.2 h) indicating the dissociation and subsequent elimination of the protein-bound probe (error bars represent SD).

Flow cytometry experiments (representative plot Fig. 2a) in serum starved H9C2 cells exposed to log-fold varying concentrations of AlexaFFA for 2 h showed that the optimal loading concentration to load 50 percent of cells was 1 µM (r^2^ = 0.96, Fig. 2b).

Furthermore*, *in vitro time course experiments showed that more than 50 percent of serum starved H9C2 cells incorporated AlexaFFA (1 µM) in 73 min (r^2^ = 0.98 Fig. 2c).

### In vivo blood kinetics

The in vivo blood kinetics of AlexaFFA showed a biexponential pattern, with an initial rapid clearance (over the first hour) followed by a plateau and slower clearance of the coupling product from the blood (Fig. 2d). These data fit a two-compartment kinetic model: the half-life of the initial fast compartment (Fig. 2e) was 0.14 h and the half-life of the slower compartment was 5.2 h (Fig. 2f).

### Ex vivo imaging

**Figure 3:**
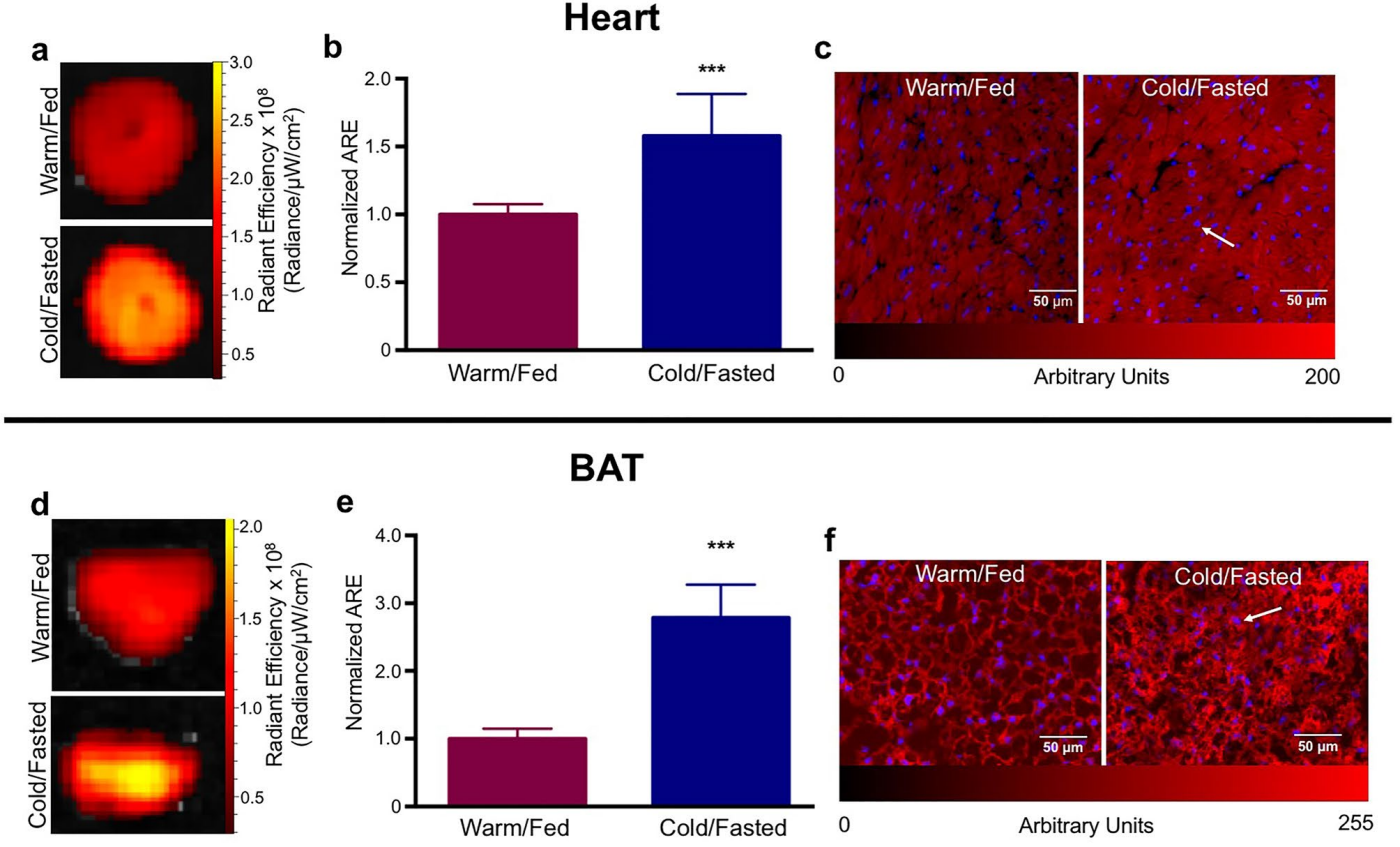
Ex vivo imaging of AlexaFFA. Fluorescence reflectance imaging of the heart in animals injected with AlexaFFA under cold/fasting or warm/fed conditions (**a**) resulting in a 58 ± 12% increase in AlexaFFA uptake in the cold/fasted hearts (**b**). Fluorescence microscopy showing increased signal intensity in cold/fasted sections recapitulating whole organ observations (**c**). A similar pattern was observed in BAT from cold/fasted vs. warm/fed animals (**d**) resulting in a 278 ± 19% increase in AlexaFFA uptake (**e**) which was also reflected in fluorescence microscopy sections (**f**). (***p < 0.001, n = 7 both groups, white arrows represent DAPI staining, *ARE* average radiant efficiency).

Stimulation of BAT and myocardial AlexaFFA uptake by cold/fasting resulted in a significant difference in uptake in both tissues compared to similarly injected warm/fed animals. Ex vivo imaging of the heart, performed 30 h post-injection, revealed a 58 ± 12% (p < 0.001) increase in signal intensity in the hearts from cold/fasted animals (Fig. 3a,b). Fluorescence intensity imaging of BAT at the same time point, post-injection, resulted in a 278 ± 19% increased signal (p < 0.001) in cold/fasted animals (Fig. 3d,e).

Fluorescence microscopy confirmed increased cellular uptake of AlexaFFA in both the heart (Fig. 3c) and BAT (Fig. 3f) in cold/fasted animals. Microscopy of BAT from cold/fasted mice also showed a denser distribution of AlexaFFA and a less vacuolated appearance than in control mice. Histology of the H & E stained sections revealed that the reason for this was the difference in the size and number of lipid droplets in non-activated (warm/fed) versus activated (cold/fasted) BAT (SI Fig. S1).

### In vivo imaging

**Figure 4:**
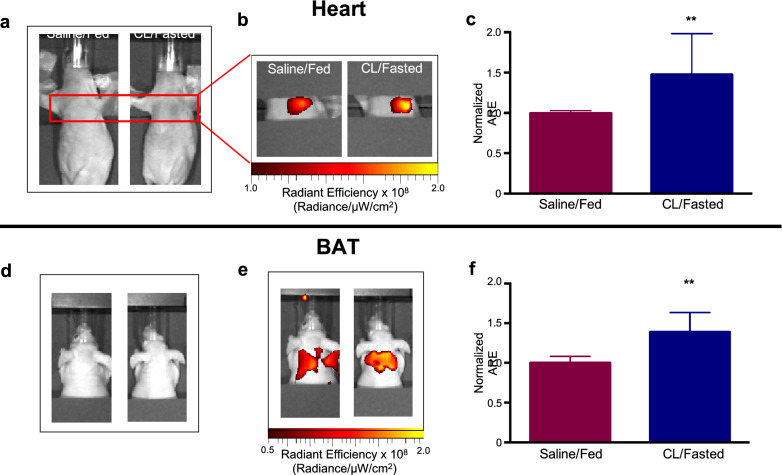
In vivo imaging of AlexaFFA. Light image of mice that were saline/fed or CL/fasted showing the thorax (**a**) and interscapular regions (**d**). Corresponding overlay fluorescence image showing increased AlexaFFA uptake in the heart (48 ± 20%; **b**,**c**) and BAT (40 ± 10%; **e**,**f**) in CL/fasted mice compared to saline/fed mice. (p < 0.01, n = 6 both groups, *ARE* average radiant efficiency).

Stimulation-mediated AlexaFFA uptake in BAT and heart could also be quantified in vivo. AlexaFFA uptake secondary to CL/fasting resulted in a 48 ± 20% increase in signal intensity (p < 0.01) in the heart 30 h post-injection compared to hearts from saline-injected/fed mice (Fig. 4b,c). In vivo fluorescence reflectance imaging of BAT at the same time point resulted in 40 ± 10% increased signal (p < 0.01) in CL/fasted mice (Fig. 4e,f). For both in vivo cardiac and BAT imaging, shielding the kidneys and bladder with black paper improved dynamic range, consistent with the renal elimination of the probe.

## Discussion

Highly metabolically active tissues such as the heart and BAT require a constant supply of oxidative fuel and thus require metabolic flexibility to function effectively. For example, the heart meets most of its energy requirements (up to 6 kg/day of ATP) through the oxidation of a variety of exogenous substrates, including fatty acids (primarily-up to 70%) and glucose (secondarily-up to 30%), with other substrates forming a smaller percentage^20,21^. In addition, dysregulation of substrate utilization and metabolism is a central pathologic feature of many disease processes^22–26^. Therefore, the ability to accurately and quantitatively assay substrate utilization has important implications for our understanding of disease pathology.

Here we describe a straightforward “click chemistry” synthesis of a novel FFA optical imaging NIR tracer. These tracers can be assembled using commercially available substrates by investigators with little chemistry background and limited access to synthetic chemistry resources. In addition, we show that AlexaFFA follows the predicted physiologic response of endogenous FFA to stimuli that increase FFA uptake in two tissues, heart and BAT, with abundant mitochondria and high energetic needs. Finally, we show that AlexaFFA can be quantified, making the in vivo assessment of FFA metabolism in deep tissues a viable pre-clinical solution.

Existing technologies such as PET and MR spectroscopy, although advantageous in certain respects, have several limitations such as relatively high cost, complex chemistry and low throughput^27^. A multiplexed optical imaging approach can overcome many of these restrictions and provide a robust pre-clinical imaging platform that can be broadly and inexpensively used by many disciplines. However, optical imaging of energy substrates has suffered from a lack practical probes for in vivo use. AlexaFFA overcomes these limitations, and the near-infrared wavelength of the probe facilitates the imaging of intact organs and deep tissues. While fluorescence tomography of NIR fluorochromes is feasible and improves the accuracy of their quantification^28,29^, the technique is not widely available and would reduce the simplicity and throughput of our optical approach. We have shown here that fluorescence reflectance imaging provides sufficient accuracy to distinguish important physiological states. It is possible, however, that more subtle differences would require fluorescence tomography, which is fully supported by AlexaFFA, to optimize their detection.

Click chemistry has simplified our capacity to synthesize molecules to track cellular level processes by enabling high-yield and broadly applicable protocols to conjugate imaging probes to biochemical substrates^30,31^. The growing library of click-compatible dyes and substrates has empowered non-chemists with capability to synthesize probes to interrogate many cellular and biochemical mechanisms in a cost-effective manner^32^. Using inexpensive, commercially available, azide and alkyne precursors, we performed efficient, copper-free click chemistry resulting in an optically detectable FFA probe (Fig. 1). Because we used excess 15-azidopentadecanoic, virtually all the Alexa Fluor 647 DIBO Alkyne was converted into the products (94% purity) as confirmed by HPLC (Fig. 1b-blue tracing). The doublet peak likely represents two product regioisomers (Fig. 1b,c), both of which are likely optically and metabolically active and required no further purification.

Furthermore, HPLC analysis suggested that a small amount (6% by-product) of the product likely reacted further with 15-azidopentadecanoic acid (Fig. 1b; peak 1685^+^ *m/z*). Although the vendor did not provide the proprietary structure or exact molecular weight for Alexa Fluor 647 DIBO Alkyne, the mass difference between the products is 241 g/mol and may represent a nucleophilic group on the Alexa Fluor 647 DIBO portion of regioisomer 1 and 2 becoming displaced by the azide group on unreacted palmitic acid azide, as shown schematically in SI Fig. S2. The mass 1685^+^ *m/z* can be predicted by the addition of palmitic acid azide (283 g/mol) and subtracting the azide group (42 g/mol). To maintain the simplicity and robustness of the chemical synthesis, we refrained from additional purification and observed no adverse biological effects as explained below. Moreover, this by-product may also be limited in the future by performing the reaction with a lower molar concentration of 15-azidopentadecanoic acid azide.

In vitro studies in a cardiac relevant cell line showed that the EC_50_ of the concentration-loading curve is approximately 1 μM, which is very similar to the concentration we aimed to achieve in the plasma following in vivo injection. The time-response curve shows a plateaued mono-exponential accumulation pattern, suggesting that the mechanism driving the cellular uptake of AlexaFFA is saturable and likely specific. The loading curve also shows that the slow elimination of AlexaFFA from the blood is vital, since complete uptake of the probe in H9C2 cells can take up to 3 h. The imaging in this study was performed greater than 5 blood half-lives after injection, well beyond this 3-h mark, optimizing the target-to-background ratio of AlexaFFA.

In vivo blood half-life measurements indicated that AlexaFFA, is cleared in two phases (Fig. 2). During the first 6 h there was a rapid clearance of the probe from the blood (half-life of 0.14 h). Given the rapidity of the clearance, this likely represents elimination of unbound AlexaFFA from the blood stream via the kidneys^33^. A slower clearance (half-life of 5.2 h) followed the initial rapid decline of AlexaFFA. Since the probe contains a FFA that likely binds to albumin and/or other plasma proteins, the slow clearance phase probably reflected the slow dissociation of the protein-bound fraction of the probe.

AlexaFluor, and its related unconjugated dyes, do not cross cell membranes on their own^34^. We hypothesized that the addition of a medium-chain free fatty acid (palmitic acid) would enable the probe to follow the kinetics of endogenous FFA. Also, we hypothesized that intracellular AlexaFFA would be retained and undergo β-oxidation. After 30 h post-injection (more than five blood half-lives), we performed fluorescence imaging, and the strong parenchymal signal of the heart and BAT, despite several washes with PBS to clear the tissue of blood, supported cellular uptake and retention of the probe. Fluorescence microscopy further confirmed the intracellular presence of AlexaFFA (Fig. 3c,f), though the fluorescence observed may reflect a combination of free fluorochrome as well as AlexaFFA inside the cell. One of the advantages of imaging in the near infrared lies in the low levels of background autofluorescence from tissues. We confirmed this to be the case in both the heart and BAT (SI Fig. S3). No signal was seen from either tissue when the mice were injected with vehicle (PBS, DMSO) that did not contain AlexaFFA.

For AlexaFFA to be a viable tool to assess FFA metabolism, it needed to follow comparable physiology to endogenous FFA. Fasting shifts metabolic substrate utilization in the heart towards FFA consumption^35,36^. Our data corroborated that AlexaFFA is a robust indicator of FFA utilization in the heart as fasting and cell starvation significantly increased probe uptake in vitro*, *ex vivo and in vivo. Cold exposure and administration of a β3 adrenergic agonist, such as CL, strongly activate BAT^37–40^ resulting in increased utilization of glucose and FFA^41^. Although BAT can use both substrates equally well, it may prefer triglycerides and FFA upon activation^42^. Both clinical and pre-clinical investigations frequently use glucose uptake with ^18^FDG PET as a common in vivo imaging modality to assess BAT activity. However, PET imaging with labeled glucose alone likely under-represents BAT substrate selection and activation. Our data, employing two different methods of BAT activation (cold and a β3 agonist), recapitulate BAT’s ability to robustly utilize FFA and more importantly demonstrate that AlexaFFA follows the predicted physiology of increased FFA uptake. In addition, we have previously shown, ex vivo, that glucose uptake could be assessed simultaneously by ^18^FDG and Cerenkov luminescence^13^.

Fluorophore adjuncts, like Alexa Fluor 647 DIBO Alkyne, may modify the pharmacokinetics of the resulting compound such that it may differ from a comparable radiolabeled probe such as C^11^-palmitate. Although we found that AlexaFFA recapitulated predicted FFA physiology, comparative studies between radiolabeled and fluorescently labeled probes may be useful to further understand the uptake kinetics of AlexaFFA. Of note, our method is adaptable since the precursor products in the current study are commercially available and it is conceivable that different length FFAs could be coupled with Alexa Fluor 647 DIBO Alkyne (or other fluorochromes) thus creating a panel of fluorescent fatty acids that could be deployed in a multiplexed optical imaging approach to assess the impact of FFA substrate chain length on metabolism.

## Conclusions

In conclusion, we present a novel NIR FFA conjugate and demonstrate that the probe accurately reflects FFA metabolism in the heart and BAT under basal and stimulated conditions. We synthesized AlexaFFA via a straightforward click chemistry approach that is readily available to standard laboratories. The probe enables high throughput optical imaging of substrate utilization and is suited for both in vitro and in vivo studies. While we only used a single probe and channel, AlexaFFA advances and refines the multiplexed optical imaging approach we have developed to study tissue energetics and metabolism.

## Supplementary information

Supplementary file1
