# Supplementary material for: A novel tracer for in vivo optical imaging of fatty acid metabolism in the heart and brown adipose tissue
Source: Sci Rep. 2020 Jul 8;10:11209. doi: 10.1038/s41598-020-68065-4 (PMC7343860; doi:10.1038/s41598-020-68065-4)

A Novel Tracer for *In Vivo* Optical Imaging of Fatty Acid Metabolism in the Heart and Brown Adipose Tissue

Short Title: An optical fatty acid tracer for *in vivo* imaging

Original Article

Marcello Panagia MD DPhil[1,2], Jing Yang PhD[3], Eric Gale PhD[3], Huan Wang PhD[3], Ivan Luptak MD PhD[1], Howard H. Chen PhD[2,3], Dakshesh Patel PhD[2], Dominique Croteau BS[1], David Richard Pimentel MD[1], Markus Michael Bachschmid PhD[4], Wilson S. Colucci MD[1], Chongzhao Ran PhD[3], David E. Sosnovik MD[2,3]

1. Cardiovascular Medicine Section, Department of Medicine, Boston University Medical Center, Boston, MA
2. Cardiovascular Research Center, Massachusetts General Hospital, Boston, MA
3. Martinos Center for Biomedical Imaging, Department of Radiology, Massachusetts General Hospital, Boston, MA
4. Vascular Biology Section, Department of Medicine, Boston University School of Medicine, Boston, MA

Correspondence:
Marcello Panagia, MD DPhil
Boston University School of Medicine
Whitaker Cardiovascular Institute
650 Albany St. Boston, MA 02118
Voice: 617-638-8071
Email: mpanagia@bu.edu

SUPPLEMENTARY FIGURE LEGENDS:

Supplementary Figure 1. H & E staining of BAT from mice under warm/fed or cold/fasted conditions. Fewer lipid droplets (vacuolated spaces) and more intense pink staining are seen in cold/fasted animals consistent with BAT activation and consumption of internal lipid stores as oxidative fuel.

Supplementary Figure 2. Schematic representing the proposed reaction of regioisomers 1,2 with additional 15-azidopentadecanoic acid producing by-products (regioisomer 3,4) which are noted as an ionized mass of 1685^{+} *m/z* in Figure 1.

Supplementary Figure 3. Representative ex vivo images in heart and BAT in warm/fed and cold/fasted conditions with either vehicle injection or AlexaFFA injection. Images show advantage of NIR probes with low levels of background autofluorescence with vehicle treatment.

SUPPLEMENTARY FIGURES:

Supplementary Figure 1:

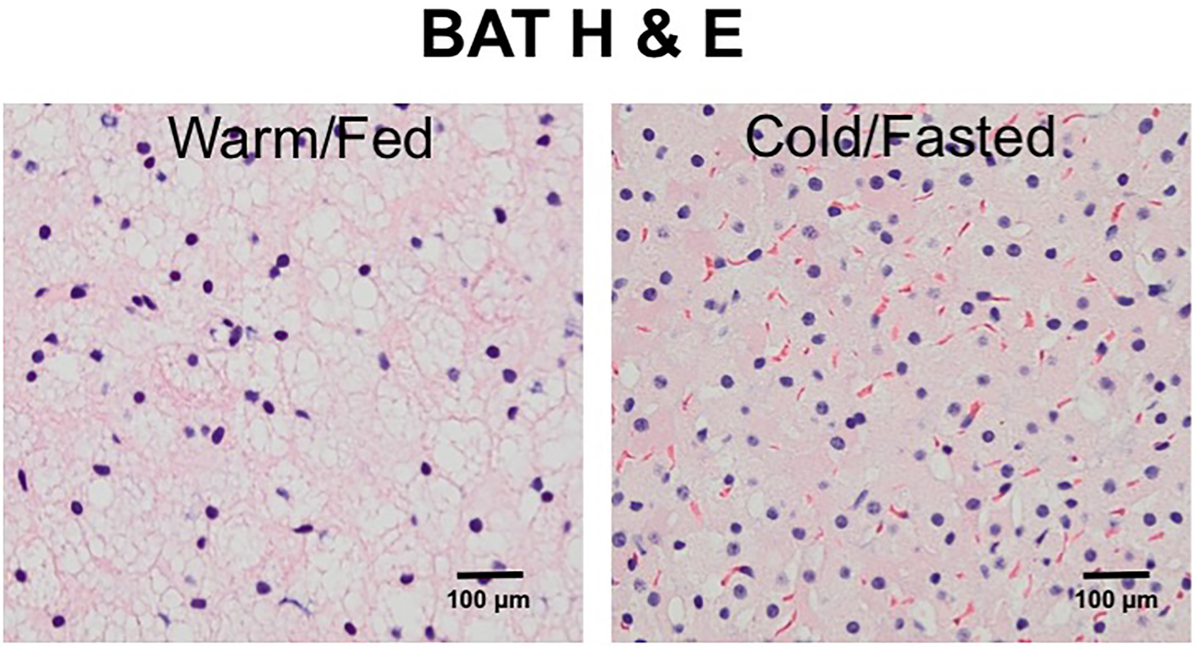

Supplementary Figure 2:

Proposed Substrates and By-Products

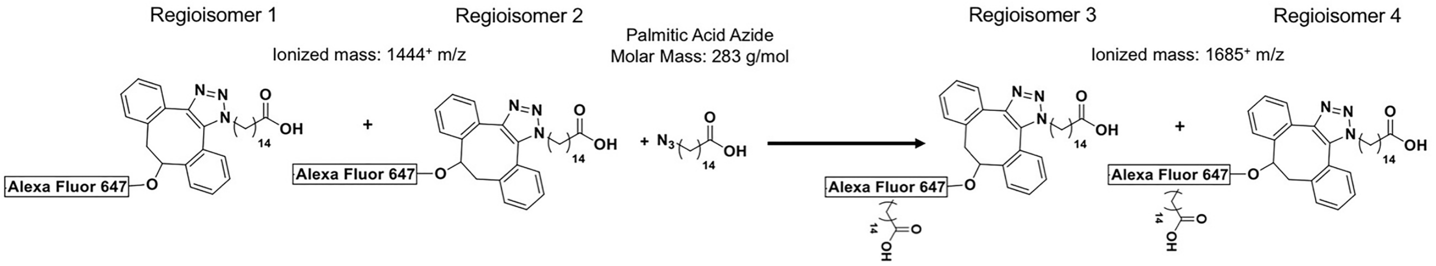

Supplementary Figure 3:

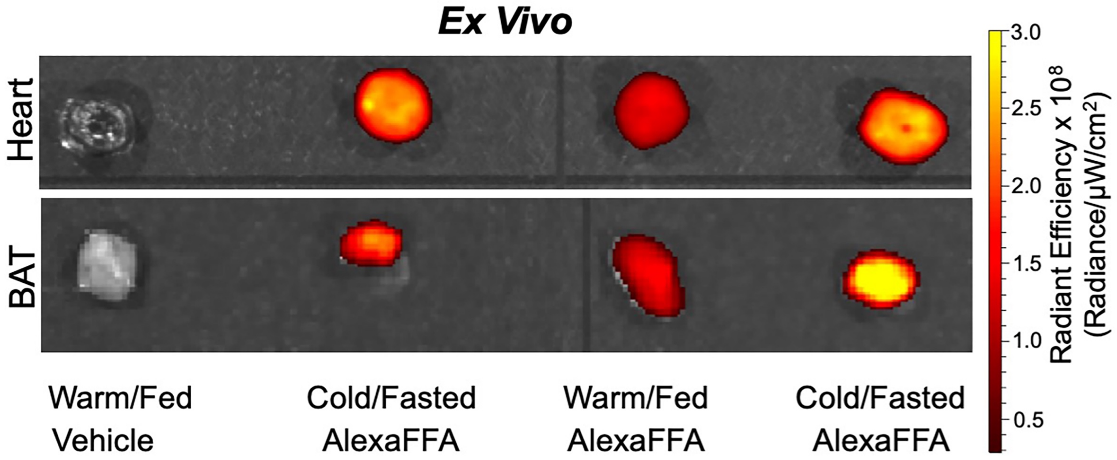

Supplement: Supplementary file 1 — Supplementary file1 [file 41598_2020_68065_MOESM1_ESM.pdf]
